# Hsp90 as a Myokine: Its Association with Systemic Inflammation after Exercise Interventions in Patients with Myositis and Healthy Subjects

**DOI:** 10.3390/ijms231911451

**Published:** 2022-09-28

**Authors:** Xiao Švec, Hana Štorkánová, Maja Špiritović, Kryštof Slabý, Sabína Oreská, Aneta Pekáčová, Barbora Heřmánková, Kristýna Bubová, Petr Česák, Haya Khouri, Gulalai Amjad, Heřman Mann, Martin Komarc, Karel Pavelka, Ladislav Šenolt, Josef Zámečník, Jiří Vencovský, Michal Tomčík

**Affiliations:** 1 Institute of Rheumatology, 128 00 Prague, Czech Republic; 2 1st Faculty of Medicine, Charles University, 121 08 Prague, Czech Republic; 3 Department of Rheumatology, 1st Faculty of Medicine, Charles University, 128 50 Prague, Czech Republic; 4 Department of Physiotherapy, Faculty of Physical Education and Sport, Charles University, 162 52 Prague, Czech Republic; 5 Department of Rehabilitation and Sports Medicine, 2nd Faculty of Medicine, Charles University, University Hospital Motol, 150 06 Prague, Czech Republic; 6 Department of Human Movement Laboratory, Faculty of Physical Education and Sport, Charles University, 162 52 Prague, Czech Republic; 7 Department of Methodology, Faculty of Physical Education and Sport, Charles University, 162 52 Prague, Czech Republic; 8 Department of Pathology and Molecular Medicine, 2nd Faculty of Medicine, Charles University, University Hospital Motol, 150 06 Prague, Czech Republic

**Keywords:** heat shock protein 90, idiopathic inflammatory myopathies, myokines, cytokines, exercise, skeletal muscles

## Abstract

Compelling evidence supports the health benefits of physical exercise on the immune system, possibly through the molecules secreted by the skeletal muscles known as myokines. Herein, we assessed the impact of exercise interventions on plasma Heat shock protein 90 (Hsp90) levels in 27 patients with idiopathic inflammatory myopathies (IIM) compared with 23 IIM patients treated with standard-of-care immunosuppressive therapy only, and in 18 healthy subjects undergoing strenuous eccentric exercise, and their associations with the traditional serum markers of muscle damage and inflammation. In contrast to IIM patients treated with pharmacotherapy only, in whom we demonstrated a significant decrease in Hsp90 over 24 weeks, the 24-week exercise program resulted in a stabilization of Hsp90 levels. These changes in Hsp90 levels were associated with changes in several inflammatory cytokines/chemokines involved in the pathogenesis of IIM or muscle regeneration in general. Strenuous eccentric exercise in healthy volunteers induced a brief increase in Hsp90 levels with a subsequent return to baseline levels at 14 days after the exercise, with less pronounced correlations to systemic inflammation. In this study, we identified Hsp90 as a potential myokine and mediator for exercise-induced immune response and as a potential biomarker predicting improvement after physiotherapy in muscle endurance in IIM.

## 1. Introduction

Although irrefutable evidence supports many health benefits of exercise, some of the underlying molecular mechanisms remains unclear. One possible mechanism is that skeletal muscles release certain molecules exerting effects on various organs and tissues, including the liver, heart, lung, skin, adipose tissues, and skeletal muscles themselves [1,2,3,4,5]. These molecules were first coined “myokines” by Pedersen et al. in 2003 [6,7], and hundreds of molecules have been identified as myokines since then [8]. Furthermore, there is increased recognition that skeletal muscle is an immunoregulatory organ that influences leukocytes and inflammation [9,10,11,12,13].

One of the potential myokines and mediators for post-exercise immune response is the Heat shock protein 90 (Hsp90). Hsp90 is an intracellular chaperone protein responsible for maintaining muscle homeostasis under stress such as exercise, and can be released into the extracellular space via either cellular damage or transport vesicles [14,15,16,17]. Additionally, Hsp90 elicits immune response via cytokines and is upregulated under acute or chronic inflammatory conditions [18,19]. In particular, our recent study demonstrated elevated systemic Hsp90 levels in patients with idiopathic inflammatory myopathies (IIM) compared with healthy controls, especially their association with muscle involvement, as well as a reduction in systemic Hsp90 levels upon standard-of-care immunosuppressive treatment [20].

Idiopathic inflammatory myopathies, also termed as myositis, constitute a heterogenous group of autoimmune conditions affecting the skeletal muscles, skin, and internal organs [21]. IIM can be classified into several subgroups: dermatomyositis (DM, including clinically amyopathic dermatomyositis (CADM)), polymyositis (PM), anti-synthetase syndrome (ASS), inclusion body myositis (IBM), immune-mediated necrotizing myopathy (IMNM), and overlap myositis (OM) [22,23,24]; however, the major complaints across different subtypes are muscle weakness and fatigue [25,26]. Despite previous concerns that exercise would exacerbate inflammation in these patients, compelling evidence has proved the opposite: exercise or physiotherapy is an effective non-pharmacological intervention to maintain and improve muscle strength and function in IIM patients [27,28,29,30], including our recent study on IIM patients after a 24-week exercise intervention [31]. These observations predicate the need for further investigation on the association between post-exercise systemic levels of myokines such as Hsp90 and inflammatory markers in order to facilitate a mechanistic understanding on how exercise interventions influence the systemic immune response.

In this study, the effect of exercise interventions on the systemic levels of Hsp90 and the association between Hsp90 levels and the traditional serum markers of muscle damage and inflammation in patients with IIM were explored. Moreover, due to the clinical relevance of exercise-induced changes in Hsp90 levels, Hsp90 levels and their association with inflammatory markers were also evaluated in healthy volunteers with acute muscle damage induced by strenuous eccentric exercise and the subsequent muscle regeneration independent of autoimmune-mediated inflammation of skeletal muscles. This control cohort was designed to mimic the clinical conditions in some IIM patients where inflammation was ameliorated by immunosuppressive therapy, but further functional improvement was precluded by the persisting muscle damage.

## 2. Results

### 2.1. Hsp90 Levels in IIM Patients: The Intervention Group (n = 27)

**Figure 1:**
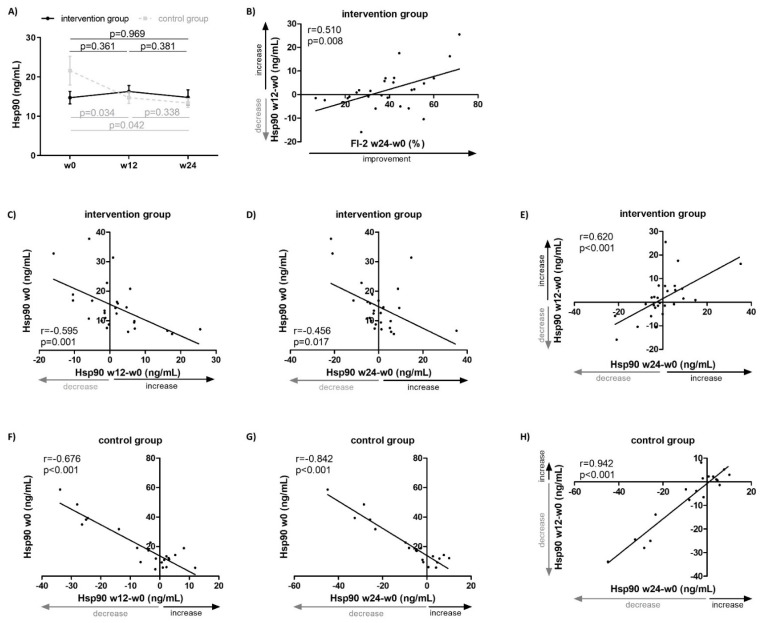
Plasma Hsp90 levels in patients with idiopathic inflammatory myopathies treated with standard-of-care pharmacotherapy (control group, CG, *n* = 23) or with additional non-pharmacological intervention (intervention group, IG, *n* = 27) for 24 weeks. (**A**) Differences in plasma Hsp90 levels over 24 weeks in both IG and CG (lines represent the mean, whiskers represent the standard error of the mean). (**B**) An increase in plasma Hsp90 levels over 12 weeks predicted an improvement in muscle endurance assessed by Functional Index-2 (FI-2) over the entire 24-week intervention. Lower baseline plasma Hsp90 levels in IG predicted an increase in plasma Hsp90 over weeks 0–12 (**C**) and 0–24 (**D**). (**E**) An increase in plasma Hsp90 levels over weeks 0–12 in IG predicted an increase in plasma Hsp90 over weeks 0–24. Higher baseline plasma Hsp90 levels in CG predicted a decrease in plasma Hsp90 over weeks 0–12 (**F**) and 0–24 (**G**). (**H**) A decrease in plasma Hsp90 levels over weeks 0–12 in CG predicted a decrease in plasma Hsp90 over weeks 0–24. W, week.

The present study is a sequel to our previously published work on a 24-week physiotherapy program assessed by primary outcomes (including Manual Muscle Testing of eight muscle groups (MMT-8) and Functional Index-2 (FI-2)) and secondary outcomes (including the Health Assessment Questionnaire (HAQ), Medical Outcomes Study 36-item Short Form Health Survey (SF-36), Fatigue Impact Scale (FIS), Beck’s Depression Inventory-II (BDI-II), and force vector area (FVA)). In that study, the intervention group (IG) of IIM patients followed a 24-week physiotherapy program in addition to the continued standard-of-care pharmacotherapy, whereas the control group (CG) was only treated with the continued standard-of-care immunosuppressive pharmacotherapy. In the IG, we observed a significant improvement (*p* < 0.05 in all) in muscle strength by 26% from baseline (assessed by MMT-8), endurance by 135% from baseline (assessed by FI-2), stability by 11% from baseline (assessed by FVA), and reduced disability by 39% from baseline (assessed by HAQ), which was clinically meaningful in a substantial proportion of patients [31]. In contrast to another recent study from our group where we described a significant decrease in plasma levels of Hsp90 from baseline to 24 weeks in the CG (baseline: 15.7 [9.8–29.5], week 24: 12.9 [9.7–16.6] ng/mL, *p* = 0.042) [20], in the present study we observed a stabilization of the plasma Hsp90 levels over 24 weeks in the IG (baseline: 12.5 [8.9–16.8], week 24: 12.0 [8.0–16.1] ng/mL, *p* = 0.969; difference between IG and CG: *p* = 0.014) (Figure 1A). Of particular interest, the change in plasma Hsp90 in the IG over the first 12 weeks was able to predict the improvement in muscle endurance (FI-2) over the entire 24-week intervention period (r = 0.510, *p* = 0.008) (Figure 1B).

A more detailed analysis on plasma Hsp90 dynamics in the IG indicated that lower baseline (week 0) plasma Hsp90 levels predicted an increase in plasma Hsp90 over weeks 0–12 (r = −0.595, *p* = 0.001) (Figure 1C) and 0–24 (r = −0.456, *p* = 0.017) (Figure 1D). Furthermore, an increase in plasma Hsp90 in the first 12 weeks of the intervention was associated with an increase over the entire 24-week intervention (r = 0.620, *p* < 0.001) (Figure 1E).

**Figure 2:**
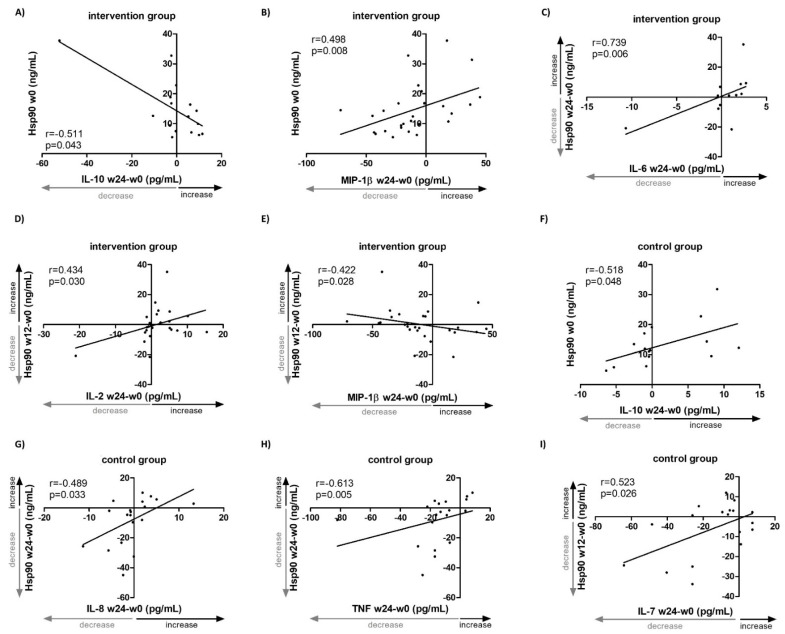
Associations of changes in systemic levels of Hsp90 and selected inflammatory cytokines/chemokines in patients with idiopathic inflammatory myopathies treated with standard-of-care pharmacotherapy (control group, CG, *n* = 23) or with additional non-pharmacological intervention (intervention group, IG, *n* = 27) for 24 weeks. Lower baseline plasma Hsp90 levels in IG predicted an increase in serum IL-10 (**A**) and a decrease in MIP-1β (**B**) over weeks 0–24. (**C**) Changes in plasma Hsp90 levels in IG over weeks 0–24 correlated positively with changes in serum IL-6 over weeks 0–24. Changes in plasma Hsp90 levels in IG over weeks 0–12 correlated positively with changes in serum IL-2 (**D**) and negatively with MIP-1β (**E**) over weeks 0–24. (**F**) Lower baseline plasma Hsp90 levels in CG predicted a decrease in serum IL-10 over weeks 0–24. Changes in plasma Hsp90 levels in CG over weeks 0–24 correlated positively with changes in serum IL-8 (**G**) and TNF (**H**) over weeks 0–24. (**I**) Changes in plasma Hsp90 levels in CG over weeks 0–12 correlated positively with changes in serum IL-7 over weeks 0–24. W, week.

To better understand the associations of the dynamics of systemic Hsp90 levels with selected inflammatory markers, the changes in selected inflammatory cytokines and chemokines observed in the IG need to be briefly described first. From baseline (week 0) to week 24, we observed a significant decrease in interleukin (IL)-7, IL-9, regulated on activation/normal T cell expressed and secreted (RANTES, also known as CCL5) and tumor necrosis factor (TNF). Lower baseline (week 0) plasma Hsp90 levels predicted an increase in IL-10 (r = −0.511, *p* = 0.043) (Figure 2A) and a decrease in MIP-1β (r = 0.498, *p* = 0.008) (Figure 2B) over weeks 0–24. Moreover, changes in Hsp90 over weeks 0–24 correlated positively with changes in IL-6 (r = 0.739, *p* = 0.006) (Figure 2C), despite only about a half of the patients having measurable levels of IL-6. Similarly, changes in Hsp90 over the first 12 weeks of intervention correlated positively with changes in IL-2 (r = 0.434, *p* = 0.030) (Figure 2D) over weeks 0–24. In addition, an increase in Hsp90 over weeks 0–12 was associated with a decrease in MIP-1β (r = −0.422, *p* = 0.028) (Figure 2E) over weeks 0–24. All changes in the plasma levels of selected cytokines and chemokines and Hsp90 over the 24-week intervention are summarized in Tables S1 and S2.

### 2.2. Hsp90 Levels in IIM Patients: The Control Group (n = 23)

In the CG of IIM patients who were only on continued standard-of-care immunosuppresive pharmacotherapy but did not undergo physiotherapy, higher baseline (week 0) plasma Hsp90 levels predicted a decrease in plasma Hsp90 over weeks 0–12 (r = −0.676, *p* < 0.001) (Figure 1F) and 0–24 (r = −0.842, *p* < 0.001) (Figure 1G). Furthermore, a decrease in plasma Hsp90 in the first 12 weeks of the intervention was associated with a decrease over the entire 24 weeks (r = 0.942, *p* < 0.001) (Figure 1H, Table S3).

Regarding inflammatory markers, in the CG we observed a significant increase in IL-2 and IL-6 and a significant decrease in IL-9, MIP-1β, MCP-1, RANTES, and TNF (Table S1); however, the analysis on IL-6 was limited by a low number of patients with detectable levels. Lower baseline (week 0) plasma Hsp90 levels predicted a decrease in IL-10 (r = 0.518, *p* = 0.048) over weeks 0–24 (Figure 2F, Table S3). Over weeks 0–24, changes in Hsp90 correlated positively with changes in IL-8 (r = 0.489, *p* = 0.033) (Figure 2G) and TNF (r = 0.613, *p* = 0.005) (Figure 2H). Similarly, the change in Hsp90 over the first 12 weeks correlated postitively with the change in IL-7 (r = 0.523, *p* = 0.026) (Figure 2I, Table S3). All changes in the plasma levels of the selected cytokines, chemokines, and Hsp90 over the 24-week standard-of-care pharmacotherapy in the CG are summarized in Tables S1 and S3.

### 2.3. Hsp90 Levels in Healthy Subjects after Exercise Intervention (n = 18)

**Figure 3:**
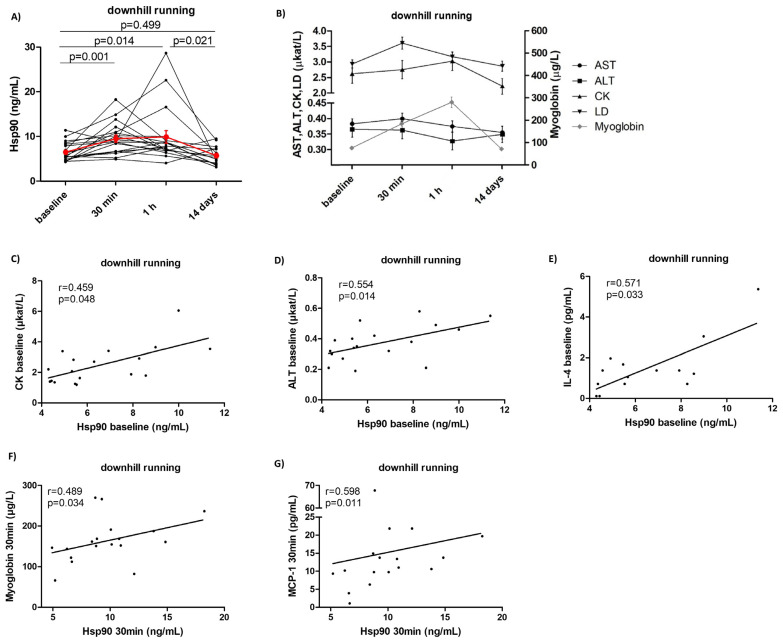
Systemic levels of Hsp90 and selected traditional markers of muscle damage and inflammation in healthy volunteers (*n* = 18) upon strenuous exercise. Systemic levels of Hsp90 (**A**) and of AST, ALT, CK, LD, and myoglobin (**B**) from baseline to 14 days after the intervention. Baseline plasma Hsp90 levels correlated positively with baseline serum levels of CK (**C**), ALT (**D**), and IL-4 (**E**). Plasma Hsp90 levels at 30 min correlated positively with serum levels of myoglobin (**F**) and MCP-1 (**G**) at 30 min. The red line (**A**) and the black/gray lines (**B**) represent the mean and the whiskers (**A**,**B**) represent the standard error of the mean.

**Figure 4:**
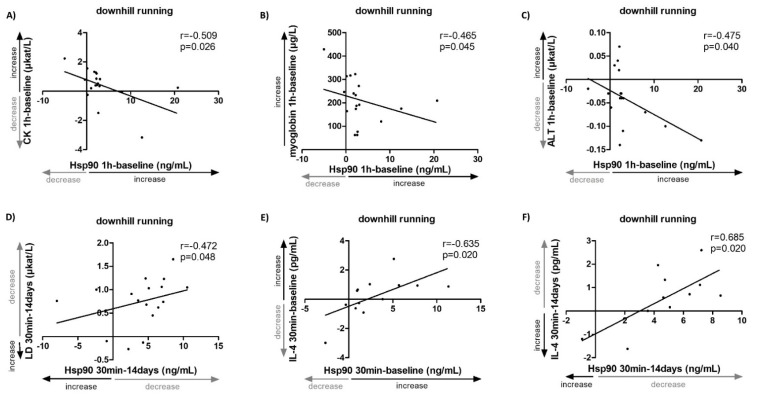
Associations of changes in systemic levels of Hsp90, traditional markers of muscle damage and inflammation in healthy volunteers (*n* = 18) upon strenuous exercise. The change in plasma Hsp90 from baseline to 1 h was inversely correlated with change from baseline to 1 h in serum levels of CK (**A**), myoglobin (**B**), and ALT (**C**). (**D**) A decrease in Hsp90 from 30 min to 14 days was associated with a decrease in LD from 30 min to 14 days. (**E**) An increase in Hsp90 from baseline to 30 min was associated with an increase in IL-4 from baseline to 30 min. (**F**) A decrease in Hsp90 from 30 min to 14 days was associated with a decrease in IL-4 from 30 min to 14 days.

In this study, the impact of strenuous exercise interventions on Hsp90 levels in 18 healthy subjects was assessed, as well as the association between Hsp90 levels and the traditional serum markers of muscle damage and inflammation. In order to achieve exercise-induced muscle damage and subsequent regeneration, we chose a downhill running protocol which involves eccentric contractions of the muscles [32]. Plasma Hsp90 levels peaked 30 min after the end of the exercise and returned to baseline levels after 14 days (baseline: 5.6 [4.8–8.4], 30 min: 9.1 [6.6–11.2], 1 h: 7.7 [6.8–9.3], 14 days: 4.9 [3.5–7.4] ng/mL, *p* = 0.005) (Figure 3A, Table S4). Similar trends in dynamics were observed in the serum levels of the traditional markers of muscle damage: alanine aminotransferate (ALT), aspartate aminotransferase (AST), creatine phosphokinase (CK), lactate dehydrogenate (LD), and myoglobin (Figure 3B), as well as MCP-1 (Table S4). No significant changes in the other selected cytokines/chemokines were observed (Table S4). Of particular interest, baseline plasma Hsp90 levels correlated positively with baseline serum levels of CK (r = 0.459, *p* = 0.048) (Figure 3C) and ALT (r = 0.554, *p* = 0.014) (Figure 3D), as well as with IL-4 (r = 0.571, *p* = 0.033) (Figure 3E). At 30 min, plasma Hsp90 correlated positively with myoglobin (r = 0.489, *p* = 0.034) (Figure 3F) and MCP-1 (r = 0.598, *p* = 0.011) (Figure 3G, Table S5). Moreover, several associations between the changes in plasma Hsp90 and the levels of selected markers of muscle damage at identical time intervals were observed. At 1 h from baseline, the change in Hsp90 was inversely correlated with the change in serum levels of CK (r = −0.509, *p* = 0.026) (Figure 4A), myoglobin (r = −0.465, *p* = 0.045) (Figure 4B), and ALT (r = −0.475, *p* = 0.040) (Figure 4C, Table S5). From 30 min to 14 days, a decrease in Hsp90 was associated with a decrease in LD (r = 0.472, *p* = 0.048) (Figure 4D, Table S5). Interestingly, the change in Hsp90 from baseline to 30 min, as well as from 30 min to 14 days, demonstrated a strong positive correlation with the change in IL-4 levels (r = 0.635, *p* = 0.020, and r = 0.685, *p* = 0.020, respectively) (Figure 4E,F); nevertheless, this observation was limited by a low number of healthy individuals with detectable levels of IL-4 (Table S5). The rest of the measured cytokines/chemokines, not listed in Tables S1 and S2 (e.g., IL-2, IL-6, IL-8 and IL-10), were undetectable in most healthy subjects of this cohort.

## 3. Discussion

In this study, Hsp90 as a myokine was identified and the dynamic changes in systemic Hsp90 levels were described in association with traditional markers of muscle damage and inflammation after exercise interventions in IIM patients and healthy volunteers after strenuous eccentric exercise. These two intervention groups exemplified two different etiologies of muscle damage: predominantly autoimmune-mediated inflammatory and exercise-induced non-inflammatory muscle damage with its subsequent repair and regeneration. In IIM patients, the change in Hsp90 plasma levels was able to predict the overall improvement in muscle endurance.

**Figure 5:**
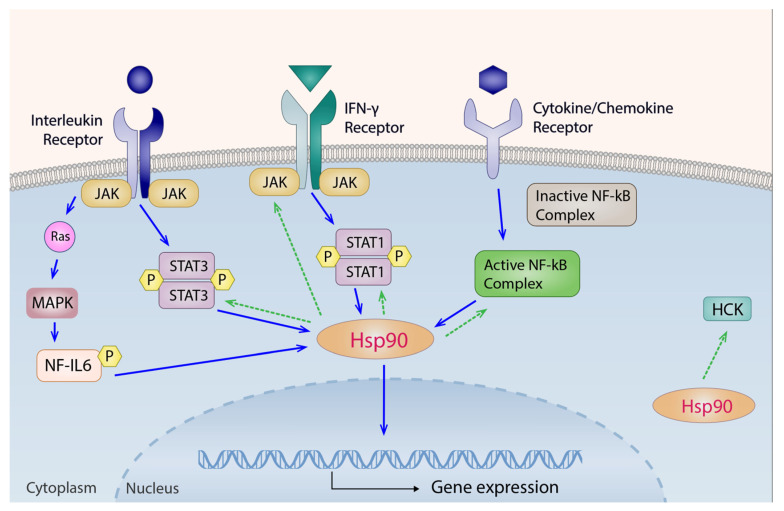
Signaling pathways of Hsp90 in the inflammatory process. Solid blue arrows indicate signaling pathways that upregulate Hsp90 expressions in the inflammatory process. IL-6 upregulates Hsp90 via both the JAK-STAT3 and the NF-IL6 pathways. INF- γ increases Hsp90 expressions through the JAK-STAT1 pathway. Other cytokines/chemokines such as TNF and IL-1 can upregulate Hsp90 by activating the NF-kB complex. Dashed green arrows denote relevant pathways influenced by Hsp90. Hsp90 binds and stabilizes JAK and STAT proteins, as well as the nonreceptor tyrosine kinase HCK, which is known to regulate innate immune response. Reciprocatively, Hsp90 is required to induce NF-kB. HCK, hematopoietic cell kinase; IL, interleukin; INF, interferon; JAK, Janus kinase; MAPK, mitogen-activated protein kinase; NF-kB, nuclear factor kappa-light-chain-enhancer of activated B cells; Ras, rat sarcoma protein, or Ras GTPase protein; STAT, signal transducer and activator of transcription.

Thus far, systemic levels of Hsp90 have never been reported in either IIM patients or healthy subjects after exercise interventions. Nonetheless, our recently published cross-sectional study demonstrated increased Hsp90 plasma levels in IIM patients compared with healthy controls, which were associated with muscle involvement, higher disease activity, and several cytokines/chemokines (IL-1b, IL-6, IL-8, IL-17, interferon γ (IFN-γ), C-X-C motif chemokine ligand 10 (CXCL10), MCP-1, MIP-1α, MIP-1β, vascular endothelial growth factor (VEGF), and platelet-derived growth factor-(PDGF)-BB) previously implicated in the pathogenesis of IIM [20,21,22,33,34,35]. In that study, a decrease in Hsp90 levels after standard-of-care immunosuppressive therapy in IIM patients with both early and established disease was described [20]. It is also worth mentioning that the longitudinal IIM cohort with established disease from that study was further analyzed in this study and served as the control cohort for the IIM IG, as originally designed in our 24-week exercise intervention study [31]. In the CG of this study, although we did not find any significant changes in the mean serum levels of LD and CK at week 24 compared to baseline after continued immunosuppressive therapy (LD: −9%, *p* = 0.897; CK: +5%, *p* = 0.154) [20], we observed a significant increase in IL-2 and IL-6 and a significant decrease in IL-9, MIP-1β, MCP-1, RANTES, and TNF. Meanwhile, systemic levels of all of these cytokines have been shown to correlate with several IIM-specific features [33,36,37,38,39,40]. Furthermore, the higher the baseline Hsp90, the larger the decrease in Hsp90 both over the first 12 and throughout the entire 24 weeks of continued immunosuppressive therapy was detected. Similarly, the extent of decrease in the first 12 weeks predicted the decrease over the entire 24-week follow-up in this IIM CG. Moreover, positive associations of changes in Hsp90 with changes in IL-7, IL-8, and TNF were detected. Relatedly, TNF was previously found to induce cleavage of Hsp90, which then potentiates apoptosis and cellular death [41]. It is apparent that the role of Hsp90 in the inflammatory process is intricate and context dependent (Figure 5). On the one hand, Hsp90 expression can be upregulated by IL-6 via the nuclear factor for IL-6 (NF-IL6) and the signal transducer and activator of transcription 3 (STAT-3) pathways [42], as well as by INF- γ via the Janus kinase (JAK)-STAT1 pathway [43]. Other cytokines/chemokines such as TNF and IL-1 can upregulate Hsp90 by activating the nuclear factor kappa-light-chain-enhancer of activated B cells (NF-kB) complex [44,45]. On the other hand, Hsp90 binds and stabilizes JAK and STAT proteins [46], as well as the hematopoietic cell kinase (HCK), which is known to regulate innate immune response [47]. Reciprocatively, Hsp90 is required to induce NF-kB and its downstream proinflammatory response [44]. These findings in the IIM CG might implicate a potential link between Hsp90 response and the aforementioned cytokines previously implicated in the pathogenesis of IIM, especially in the autoimmune-mediated muscle inflammation and/or damage and its subsequent repair upon standard-of-care immunosuppressive therapy.

As demonstrated in our previous study, our 24-week exercise intervention for IIM patients was not only safe (no patient dropped out of the program, no increase in traditional serum markers for muscle damage was observed, no increased systemic or local expression of selected pro-inflammatory markers was reported), but also proved effective, evidenced by significantly improved muscle strength, endurance, stability, and reduced disability [31]. As a continuation of the previous study, Hsp90 levels were stabilized over the 24-week intervention in the IG of this study, compared with decreased Hsp90 levels in the CG. There was even a slight numerical increase in Hsp90 for the first 12 weeks of intervention, albeit not statistically significant. Of particular interest, at the individual level, the extent of increase in plasma Hsp90 over the first 12 weeks predicted the rate of improvement in muscle endurance (FI-2) over the entire 24-week intervention. Furthermore, in the IG, we observed a significant decrease in IL-7, IL-9, RANTES, and TNF. Some of these findings are corroborated by previous studies in non-IIM conditions where post-exercise decrease in TNF and RANTES were reported [48,49,50,51,52]. We did not observe any statistically significant changes in MIP-1β, although one previous study demonstrated elevated MIP-1β levels after resistance exercise in the elderly [53]. Analogous to the CG, in the IG, the lower the baseline Hsp90 levels, the more prominent the increase in Hsp90 over the first 12 weeks and the entire 24 weeks of intervention was observed. The changes of Hsp90 over the first 12 weeks of intervention predicted changes over the entire 24-week intervention. In the IG, associations of changes in Hsp90 with several cytokines/chemokines were also observed, particularly with IL-2 and MIP-1β. In accordance with our findings, previous studies demonstrated that IL-2 induces Hsp90 synthesis in vitro [54] and inhibition of Hsp90 reduces IL-2 levels [55]. Moreover, both IL-6 and IL-10 could upregulate Hsp90 via the STAT signaling pathways according to a study by Ripley et al. [56]. Our findings in the IIM IG demonstrate that at the group level, functional improvement (predominantly in muscle endurance and strength) was accompanied by a stabilization of plasma Hsp90 levels after 24 weeks of exercise in addition to the standard-of-care immunosuppressive therapy. This is contrasted by a decrease in Hsp90 levels in the IIM CG with immunosuppressive therapy only. At the individual level, in the IIM IG, we identified a potential link between the changes in Hsp90 and muscle endurance and the aforementioned cytokines/chemokines implicated previously either in the pathogenesis of IIM or in the exercise-induced changes observed in non-IIM conditions.

For further insight into the skeletal muscle homeostasis in the context of cellular damage, an additional assessment was carried out to explore whether the post-exercise response is similar in healthy volunteers devoid of any autoimmune-mediated systemic and local inflammation. This cohort of healthy volunteers was designed to mimic part of the clinical disease course of muscle damage and subsequent regeneration in IIM patients, especially when systemic and/or local inflammation was ameliorated by immunosuppressive agents but the muscle damage persisted, precluding further improvement with pharmacotherapy only [31]. This condition was achieved in healthy volunteers by a strenuous eccentric exercise/downhill running protocol. In order to maintain consistency, the same methods were used for analysis on this healthy cohort, despite the potential limitations, especially since most of the inflammatory cytokines/chemokines in healthy volunteers were hardly detectable. After all, several inflammatory cytokines/chemokines were detected in this cohort. The timing of blood sampling (0 min, 30 min, 1 h, 14 days after the exercise intervention) was determined by existing literature [57,58,59,60] and our preliminary results from traditional serum markers of muscle damage at baseline, 30 min, 1, 3, 6, 12, 24, 48, 72, and 96 h, 7 and 14 days after the intervention. A significant increase in plasma Hsp90 levels was observed, peaking at 30 min and 1 h after the intervention and returning to baseline levels after 14 days. However, one study by Shastry et al. demonstrated no difference in Hsp90 protein levels assessed in the supernatants of isolated leukocytes in healthy volunteers 15 and 24 h after moderate exercise [61]. Similar dynamics were observed in serum levels of LD and myoglobin. Nevertheless, the other serum markers for muscle damage (AST, ALT, and CK) did not exhibit the same pattern, which might be due to the selected timing of sample collection, and we might have missed the peaks in between the times we were able to take blood samples. Moreover, positive associations of Hsp90 levels with CK and ALT at baseline were observed, as well as a positive association between Hsp90 levels and myoglobin at 30 min. Counterintuitively, while the traditional biomarkers for muscle damage (myoglobin, CK, and ALT) are more or less elevated at 1 h after the intervention, they are inversely correlated with changes in Hsp90 levels. Meanwhile, a decrease in myoglobin, CK, and ALT with increased Hsp90 levels after 1 h at the individual level was observed. This could be explained by the protective role of Hsp90 for muscle fibers in maintaining homeostasis, hence mitigating muscle damage. This is consistent with previous studies by De Paepe et al., where Hsp90 and Hsp70 were upregulated in both dystrophic and IIM muscle fibers and were speculated to be myoprotective [62,63]. Another explanation for the inverse correlation between changes in CK and Hsp90 could lie in the polymorphism of CK activity in response to eccentric contractions of the muscles, possibly due to genetic differences and pre-exercise conditions [64]. Nonetheless, changes in Hsp90 levels correlated positively to LD when returning to baseline from 30 min to 14 days after the intervention. Meanwhile, no significant differences in the selected cytokines/chemokines assessed in the cohort of healthy volunteers were revealed. It is also worth noting that MCP-1 levels peaked at 30 min and returned to baseline after 14 days post-exercise, with a significant decrease from 1 h to 14 days. In contrast, Troseid et al. observed a decrease in MCP-1 levels 12 weeks after endurance and strength training in subjects with metabolic syndrome [65], but did not report any intermediate changes before 12 weeks. Additionally, we detected positive associations between Hsp90 levels and MCP-1 and IL-4. Interestingly, both MCP-1 and IL-4 have been implicated in the pathogenesis of IIM [21,22,33,34,35,66,67,68]. Nonetheless, in most cases, inflammatory cytokines/chemokines are not systemically upregulated in healthy volunteers following similar protocols [48,69,70,71,72].

One of the major strengths of this study is the two control groups, including both IIM patients and healthy volunteers. Another key advantage is that our data were derived from real clinical practice, with unprecedented cohort size of IIM patients and thorough analysis of the clinical outcomes [73,74,75,76]. Furthermore, the sample size of our CG with 18 healthy volunteers is adequate, considering each participant followed a strict detailed protocol, followed by blood sampling at the pre-selected time intervals.

Despite compelling evidence, several challenges and limitations must be addressed. First off, the size of our IIM cohorts is relatively small, which is partially because IIM is a rare condition. Moreover, in the IIM IG, we could not distinguish between the effects of exercise and the effects of pharmacotherapy. The background pharmacotherapy in both IIM cohorts also precluded us from any direct assessment for causality. Furthermore, even though at the individual level in the IIM IG, significant associations of Hsp90 levels with the serum markers of systemic inflammation were detected, we demonstrated no significant changes at the group level, probably due to the low number of patients and the heterogeneity of clinical features of IIM patients. Thus, all reported associations of Hsp90 with cytokines/chemokines need to be interpreted with caution. Another limitation of this study is the differential expressions of chemokines in different sexes [77] and subtypes of IIM [78], so dividing our IIM cohorts and separately analyzing each subtype might render different results. However, the practicality of such classification was hindered by the rarity of this disease, and the relatively low number of patients due to the strict inclusion/exclusion criteria and protocol. Further studies with larger cohorts are warranted, since a mechanistic understanding of how exercise influences the immune processes could significantly inform and refine clinical care for IIM patients.

## 4. Materials and Methods

### 4.1. Participants and Experimental Design

The healthy runner cohort consisted of 18 healthy Caucasian volunteers (9 females; mean age: 26.2 years) who followed a downhill running protocol, which involved eccentric contraction of muscle fibers and mechanical damage to muscle cells. Plasma samples were collected at baseline, 30 min, 1 h, and 14 days after the downhill run.

For the evaluation of Hsp90 levels in IIM patients after exercise interventions, 50 IIM patients were consecutively recruited from 2007 at the Institute of Rheumatology in Prague and were divided into two groups. The IG includes 27 Caucasian IIM patients (median disease duration: 6.0 years) with ongoing standard-of-care pharmacological treatment and underwent a 24-week intensive physiotherapy intervention comprising a supervised, personalized training program performed twice a week for one hour, focused on activities of daily living and strength and stability training (clinical trial registration No. ISRCTN35925199); the detailed inclusion and exclusion criteria, baseline demographic and clinical characteristics, training protocol, and primary and secondary outcomes were published elsewhere [31]. Plasma samples were obtained at baseline and after 12 and 24 weeks of this physiotherapy intervention.

The CG includes 23 Caucasian IIM patients (median disease duration: 2.8 years) with ongoing standard-of-care pharmacological treatment but did not follow any exercise intervention. Plasma samples were also obtained at baseline and after 12 and 24 weeks of follow-up. Similarly to IG, further details on patients in CG are available elsewhere [31].

Written informed consent was obtained from all participants. This study was approved by the Ethics Committee of the Institute of Rheumatology in Prague and the Department of Rehabilitation and Sports Medicine, 2nd Faculty of Medicine, Charles University and University Hospital Motol in Prague. All procedures were carried out in accordance with the relevant guidelines and regulations.

### 4.2. Downhill Running Protocol

In total, 18 subjects were recruited for the downhill running cohort. Exclusion criteria included any serious diseases, a history of musculoskeletal, rheumatic, or autoimmune disease, any disabling medical conditions one month preceding the initial stress test, and any medication or treatment regimen. Subjects were asked to maintain regular physical activity and to avoid activities involving eccentric muscle contractions for four weeks before the initial test, and to refrain from vigorous or endurance exercise two days before the initial test and throughout the experiment. All participants were examined for musculoskeletal conditions prior to the initial test. Subjects with extremely high or low aerobic capacity (Z-scores below −2 or above 2) were excluded.

This was an interventional study on young healthy volunteers without a control group. Downhill running with moderate intensity was used because it is not physically demanding, induces muscle damage and soreness, and is safe with a duration of 20 to 45 min (rhabdomyolysis or acute kidney injury was not reported in the literature) [79].

Subjects underwent two exercise stress tests separated by seven days. Both tests were performed on a motor-driven treadmill (Valiant Plus (P/N 932902) with Rear Elevation −10% mechanical, Valiant, Lode, Netherlands). The first test was a maximal incremental test and was used to estimate the aerobic capacity and target exercise intensity for the second test. It was carried out without inclination (slope 0%), with continuous monitoring of electrocardiogram (BTL-08 LC ECG, BTL Industries Ltd., Stevenage, UK), heart rate, and respiratory indices (Oxycon Pro with an electrochemical oxygen sensor, CareFusion, Hoechberg, Germany): ventilation, oxygen consumption (VO2), carbon dioxide expenditure (VCO2), and respiratory exchange ratio (RER). Maximal test for VO2max assessment was defined as the subjects’ maximal effort (the Borg Rating of Perceived Exertion scale (RPE) > 18), heart rate above the lower limit of population reference values, and RER ≥ 1.12. The second test was designed as the intervention itself. It was carried out at a constant speed corresponding to the intensity of 60% of VO2max, with a negative slope (−10%) for 45 min. Heart rate and VO2 were measured 10 and 30 min after the test started to ensure target intensity. Before and after the intervention, a visual analog scale (VAS, 0–10) was used to record the perceived muscle pain and general fatigue. Subjects with pain or fatigue >2 were excluded. The timing of blood sampling (baseline, 30 min, 1 h, and 14 days after the intervention itself) were based on literature [57,58,59,60] and on the preliminary test results from the dynamics of AST, ALT, CK, LD, and myoglobin at the following times: baseline, 30 min, 1, 3, 6, 12, 24, 48, 72, and 96 h, 7 and 14 days after the intervention.

### 4.3. Laboratory Analysis

Hsp90 levels were measured with a commercially available ELISA kit (eBioscience, Vienna, Austria) according to the instructions. The following cytokines/chemokines were quantified by a commercially available Bio-Plex Pro^TM^ human Cytokine 27-plex Assay (BIO-RAD, Hercules, CA, USA): IL-1β, IL-1Ra, IL-2, IL-4, IL-5, IL-6, IL-7, IL-8, IL-9, IL-10, IL-12 (p70), IL-13, IL-15, IL-17A, eotaxin, fibroblast growth factor (FGF) basic, granulocyte colony-stimulating factor (G-CSF), granulocyte-macrophage colony-stimulating factor (GM-CSF), interferon-γ (IFN-γ), CXCL10, MCP-1 (CCL2), MIP-1α (CCL3), MIP-1β (CCL4), PDGF-BB, RANTES (CCL5), TNF, and VEGF. The absorbance of the Bio-Plex Pro^TM^ human Cytokine 27-plex Assay was assessed by Luminex BIO-PLEX 200 System (Bio-Rad, Hercules, CA, USA). All samples were quantified in duplicate, and the averaged values were used. The analysis on serum levels of traditional markers of inflammation (C-reactive protein (CRP)), muscle damage (AST, ALT, CK, LD, and myoglobin), and the detection of antinuclear antibodies (ANA) and myositis specific antibodies (MSA) and myositis associated antibodies (MAA) were performed as described elsewhere [20,31].

### 4.4. Statistical Analysis

All analyses were performed using SPSS version 25 (SPSS, Inc., Chicago, IL, USA). Figures were plotted using GraphPad Prism 5 (version 5.02; GraphPad Software, La Jolla, CA, USA) and Adobe Illustrator 2022 (version 26.5; Adobe Inc., San Jose, CA, USA). All variables were assessed for normality with Kolmogorov–Smirnov and Shapiro–Wilk tests. Comparisons between groups were performed by Chi-square for categorical variables, t-test for continuous variables with normal distribution, or Mann–Whitney U test for continuous variables with skewed distribution. The bivariate relationships were assessed using the Spearman or Pearson correlation coefficient. Repeated measurements were assessed by one-way repeated ANOVA followed by post hoc comparisons. Inter-group analysis on differences in repeated measurements between the IG and CG of IIM patients were assessed by two-way repeated (group × time) ANOVA. Data are presented as median (inter-quartile range) unless stated otherwise, and *p* < 0.05 was considered statistically significant.

## 5. Conclusions

This study assessed the impact of exercise interventions on Hsp90 levels in 27 patients with IIM and 18 healthy subjects, as well as the association between Hsp90 levels and the traditional serum markers of muscle damage and inflammation. The observations presented here along with our previous work suggest that the effect of exercise on inflammatory markers could be mediated by Hsp90, but further research is warranted. Moreover, plasma Hsp90 could become a potential novel biomarker to predict improvement in muscle endurance in IIM patients following physiotherapy.

## Data Availability

Individual anonymized data will not be shared. Pooled study data, protocol, or statistical analysis plans can be shared.

## Supplementary Materials

The supporting information can be downloaded at: https://www.mdpi.com/article/10.3390/ijms231911451/s1.

## Abbreviations

ALT	Alanine aminotransferase
ANA	Antinuclear antibodies
ASS	Anti-synthetase syndrome
AST	Aspartate aminotransferase
BDI-II	Beck’s Depression Inventory-II
CADM	Clinically amyopathic dermatomyositis
CG	Control group
CK	Creatine phosphokinase
CRP	C-reactive protein
CXCL10	C-X-C motif chemokine ligand 10, also known as IP-10
DM	Dermatomyositis
ELISA	Enzyme-linked immunosorbent assay
ESR	Erythrocyte sedimentation rate
FGF	Fibroblast growth factor
FI-2	Functional Index-2
FIS	Fatigue Impact Scale
FVA	Force vector area
G-CSF	Granulocyte colony-stimulating factor
GM-CSF	Granulocyte-macrophage colony-stimulating factor
HAQ	Health Assessment Questionnaire
HCK	Hematopoietic cell kinase
Hsp90	Heat shock protein 90
IBM	Inclusion body myositis
IFN	Interferon
IG	Intervention group
IIM	Idiopathic inflammatory myopathies
IL	Interleukin
IMNM	Immune-mediated necrotizing myopathy
JAK	Janus kinase
LD	Lactate dehydrogenase
MAA	Myositis associated antibodies
MAPK	Mitogen-activated protein kinase
MCP-1	Monocyte chemoattractant protein-1, also known as CCL2
MIP-1α	Macrophage inflammatory protein-1α, also known as CCL3
MIP-1β	Macrophage inflammatory protein-1β, also known as CCL4
MMT-8	Manual Muscle Testing of eight muscle groups
MSA	Myositis specific antibodies
NF-kB	Nuclear factor kappa-light-chain-enhancer of activated B cells
OM	Overlap myositis
PDGF	Platelet-derived growth factor
PM	Polymyositis
RANTES	Regulated on activation/normal T cell expressed and secreted, or CCL5
Ras	Rat sarcoma protein, or Ras GTPase protein
SF-36	Medical Outcomes Study 36-item Short Form Health Survey
STAT	Signal transducer and activator of transcription
TNF	Tumor necrosis factor
VEGF	Vascular endothelial growth factor
w	weeks
