# Supplementary material for: Hsp90 as a Myokine: Its Association with Systemic Inflammation after Exercise Interventions in Patients with Myositis and Healthy Subjects
Source: Int J Mol Sci. 2022 Sep 28;23(19):11451. doi: 10.3390/ijms231911451 (PMC9569475; doi:10.3390/ijms231911451)
Supplement: Supplementary file 1 [file ijms-23-11451-s001.zip › ijms-1909051-supplementary.pdf]

Supplementary Tables

Table S1. Plasma levels of selected cytokines and chemokines in patients with IIM

**Parameter (pg/mL)**	**Group**	**Week 0**	**Week 24**	**p-value**	**Number of subjects**
**IL-2**	Intervention	2.28 (1.62-4.17)	3.79 (1.81-8.53)	0.119	24
	Control	3.04 (2.66-5.30)	6.53 (3.79-8.55)	**0.022**	21
**IL-4**	Intervention	1.79 (1.11-2.44)	2.07 (1.62-2.38)	0.989	27
	Control	2.28 (1.79-3.20)	2.33 (1.93-2.84)	0.782	21
**IL-6**	Intervention	0.57 (0.28-1.85)	2.27 (0.69-3.01)	0.195	12
	Control	0.86 (0.28-2.70)	2.67 (1.10-3.98)	**0.043**	11
**IL-7**	Intervention	20.71 (12.23-26.00)	6.92 (4.57-11.69)	**0.018**	9
	Control	15.18 (2.59-23.30)	9.98 (8.22-14.05)	0.367	12
**IL-8**	Intervention	4.63 (3.07-6.57)	5.61 (4.36-7.92)	0.479	27
	Control	7.74 (5.80-11.61)	6.86 (6.02-9.05)	0.251	21
**IL-9**	Intervention	100.4 (94.8-127.2)	81.6 (68.2-87.6)	**<0.001**	27
	Control	111.6 (96.2-120.3)	85.3 (68.2-96.3)	**<0.001**	21
**IL-10**	Intervention	3.03 (1.71-4.37)	10.41 (1.58-11.67)	0.175	11
	Control	2.37 (1.39-5.04)	9.99 (1.35-11.67)	0.320	11
**MIP-1β**	Intervention	103.1 (93.3-108.8)	96.9 (75.8-106.8)	0.074	27
	Control	101.9 (89.5-116.2)	89.9 (79.9-103.5)	**0.007**	21
**MCP-1**	Intervention	49.4 (34.1-74.7)	37.4 (21.2-68.6)	0.079	26
	Control	48.2 (32.2-117.5)	32.1 (24.2-39.5)	**0.006**	21
**RANTES**	Intervention	9712 (7313-13060)	7421 (4124-10446)	**0.001**	27
	Control	9649 (6515-13266)	5991 (3992-9703)	**0.007**	21
**TNF**	Intervention	43.8 (38.7-56.4)	31.9 (27.0-43.4)	**0.002**	27
	Control	48.8 (46.9-58.3)	40.7 (33.3-43.9)	**<0.001**	21

Acronyms: Data are presented as median (interquartile range). Data with normal distribution were assessed using paired t-test. Data with non-normal distribution were assessed using Wilcoxon signed rank test. Statistically significant differences ($p<0.05$) are marked in bold. IIM, idiopathic inflammatory myopathies; IL, interleukin; MCP-1, monocyte chemoattractant protein 1 (CCL2); MIP-1β, macrophage inflammatory protein 1 beta (CCL4); RANTES, regulated on activation/normal T cell expressed and secreted (CCL5); TNF, tumor necrosis factor.

Table S2. Pearson's or Spearman's correlation coefficients of plasma levels of Hsp90 and inflammatory cytokines/chemokines in IIM patients (intervention group; n=27)

Parameter		Hsp90 w0	Hsp90 w24-w0	Hsp90 w12-w0	IL-2 w24-w0	IL-4 w24-w0	IL-6 w24-w0	IL-7 w24-w0	IL-8 w24-w0	IL-9 w24-w0	IL-10 w24-w0	MIP-1β w24-w0	MCP-1 w24-w0	RANTES w24-w0	TNF w24-w0
Hsp90 w0	r		-0.456	-0.595	-0.212	-0.159	-0.196	-0.043	-0.234	0.319	-0.511	0.498	-0.054	0.237	-0.164
	p		**0.017**	**0.001**	0.309	0.426	0.541	0.850	0.239	0.105	**0.043**	**0.008**	0.792	0.234	0.413
	n		27	27	25	27	12	22	27	27	16	27	26	27	27
Hsp90 w24-w0	r	-0.456		0.620	0.368	0.224	0.739	0.169	0.058	-0.242	0.447	-0.295	-0.024	-0.247	-0.068
	p	**0.017**		**<0.001**	0.071	0.262	**0.006**	0.449	0.775	0.223	0.083	0.771	0.906	0.214	0.737
	n	27		27	25	27	12	22	27	27	16	27	26	27	27
Hsp90 w12-w0	r	-0.595	0.620		0.434	0.144	0.494	-0.055	0.132	-0.154	0.497	-0.422	-0.087	-0.308	0.083
	p	0.001	**<0.001**		**0.030**	0.472	0.103	0.807	0.513	0.442	**0.050**	**0.028**	0.671	0.118	0.681
	n	27	27		25	27	12	22	27	27	16	27	26	27	27

Acronyms: Data with normal distribution were assessed using Pearson's correlation coefficient (r). Data with non-normal distribution were assessed using Spearman's correlation coefficient (r). Statistically significant correlations ($p<0.05$) are marked in bold. Hsp90, heat shock protein 90; IIM, idiopathic inflammatory myopathies; IL, interleukin; MCP-1, monocyte chemoattractant protein 1 (CCL2); MIP-1β, macrophage inflammatory protein 1 beta (CCL4); n, number of samples analyzed; RANTES, regulated on activation/normal T cell expressed and secreted (CCL5); TNF, tumor necrosis factor; w, week.

Table S3. Pearson's or Spearman's correlation coefficients of plasma levels of Hsp90 and inflammatory cytokines/chemokines in IIM patients (control group; n=23)

Parameter		Hsp90 w0	Hsp90 w24-w0	Hsp90 w12-w0	IL-2 w24-w0	IL-4 w24-w0	IL-6 w24-w0	IL-7 w24-w0	IL-8 w24-w0	IL-9 w24-w0	IL-10 w24-w0	MIP-1β w24-w0	MCP-1 w24-w0	RANTES w24-w0	TNF w24-w0
Hsp90 w0	r		-0.842	-0.676	-0.108	-0.046	-0.077	-0.280	-0.107	0.111	0.518	0.082	-0.184	-0.077	-0.206
	p		**<0.001**	**<0.001**	0.625	0.835	0.812	0.245	0.628	0.615	**0.048**	0.709	0.401	0.727	0.347
	n		19	22	23	23	12	19	23	23	15	23	23	23	23
Hsp90 w24-w0	r	-0.842		0.942	0.262	0.447	0.033	0.461	0.489	0.226	-0.184	0.014	0.400	0.020	0.613
	p	**<0.001**		**<0.001**	0.278	0.054	0.932	0.062	**0.033**	0.352	0.548	0.955	0.089	0.934	**0.005**
	n	19		18	19	19	12	19	19	19	15	19	19	19	19
Hsp90 w12-w0	r	-0.676	0.942		0.265	0.224	0.230	0.523	0.262	0.197	-0.257	0.107	0.117	-0.021	0.352
	p	**<0.001**	**<0.001**		0.234	0.317	0.472	**0.026**	0.239	0.379	0.375	0.637	0.604	0.924	0.108
	n	22	18		22	22	12	18	22	22	15	22	22	22	22

Acronyms: Data with normal distribution were assessed using Pearson's correlation coefficient (r). Data with non-normal distribution were assessed using Spearman's correlation coefficient (r). Statistically significant correlations (p<0.05) are marked in bold. Hsp90, heat shock protein 90; IIM, idiopathic inflammatory myopathies; IL, interleukin; MCP-1, monocyte chemoattractant protein 1 (CCL2); MIP-1β, macrophage inflammatory protein 1 beta (CCL4); n, number of samples analyzed; RANTES, regulated on activation/normal T cell expressed and secreted (CCL5); TNF, tumor necrosis factor; w, week.

Table S4. Systemic levels of Hsp90, markers of muscle damage, CRP, and selected cytokines/chemokines in healthy individuals after downhill running

Parameter	Baseline (n=18)	30 min (n=18)	1 hour (n=18)	14 days (n=18)	p^{12};p^{13}; p^{14}; p^{24}; p^{34}; p*
**Hsp90, ng/mL**	5.61 (4.83-8.35)	9.06 (6.63-11.23)	7.71 (6.84-9.29)	4.92 (3.45-7.39)	**0.001**; **0.014**; 0.499; **0.002**; **0.021; 0.005**
**CK, μkat/L**	2.14 (1.43-3.39)	2.51 (1.84-3.27)	2.52 (1.85-3.86)	1.96 (1.48-2.63)	0.611; 0.146; 0.340; 0.160; **0.041;** 0.176
**LD, μkat/L**	2.91 (2.55-3.42)	3.57 (3.18-3.93)	3.16 (2.71-3.76)	2.73 (2.52-3.01)	**0.002;** 0.105; 0.681; **<0.001;** 0.064; **<0.001**
**Myoglobin, μg/L**	52.1 (43.8-58.2)	157.9 (138.3-188.2)	246.4 (194.8-337.5)	48.2 (44.3-57.4)	**<0.001; <0.001;** 0.078; **<0.001**; **<0.001; <0.001**
**AST, μkat/L**	0.39 (0.31-0.42)	0.39 (0.35-0.44)	0.38 (0.31-0.44)	0.35 (0.32-0.38)	0.406; 0.644; 0.254; **0.031;** 0.396; 0.176
**ALT, μkat/L**	0.37 (0.29-0.47)	0.35 (0.27-0.47)	0.32 (0.23-0.45)	0.37 (0.25-0.42)	0.832; **0.009;** 0.503; 0.603; 0.399; 0.243
**CRP, mg/L**	0.55 (0.31-1.32)	0.42 (0.22-0.75)	0.35 (0.19-0.56)	0.47 (0.26-0.70)	0.127; 0.156; 0.074; 0.501; 0.272; 0.227
**IL-4, pg/mL †**	1.29 (0.71-1.74)	1.37 (1.05-2.24)	1.05 (0.71-1.96)	1.05 (0.71-1.67)	0.339; 0.635; 0.844; 0.288; 0.128; 0.512
**IL-7, pg/mL †**	3.92 (1.27-6.19)	6.19 (3.29-11.7)	3.92 (1.27-10.24)	7.26 (3.92-12.19)	0.362; 0.892; 0.219; 0.641; 0.844; 0.857
**IL-9, pg/mL**	83.5 (52.1-104.3)	85.3 (77.0-101.3)	78.2 (63.5-97.6)	76.8 (67.7-97.3)	0.568; 0.638; 0.730; 0.424; 0.784; 0.728
**MIP-1β, pg/mL**	175.5 (107.6-216.7)	175.2 (143.8-213.4)	167.5 (115.9-206.6)	143.0 (130.1-211.9)	0.706; 0.457; 0.584; 0.392; 0.897; 0.683
**MCP-1, pg/mL †**	9.3 (4.6-13.3)	11.0 (9.5-17.3)	10.6 (8.7-14.7)	7.6 (4.9-9.8)	0.064; 0.083; 0.202; **0.016; <0.001; 0.005**
**RANTES, pg/mL**	3789 (2370-7996)	4093 (3230-5896)	4129 (2530-5269)	3801 (2326-6807)	0.601; 0.360; 0.632; 0.631; 0.728; 0.675
**TNF, pg/mL**	26.3 (15.9-29.5)	28.9 (23.1-34.2)	26.3 (22.5-31.9)	28.9 (23.8-37.5)	0.078; 1.000; 0.256; 0.817; 0.242; 0.458

Acronyms: Data are presented as median (interquartile range). Statistically significant differences (p<0.05) are marked in bold: p^{12}, baseline vs 30 min; p^{13}, baseline vs 1 hour; p^{14}, baseline vs 14 days; p^{24}, 30 min vs 14 days; p^{34}, 1 hour vs 14 days; p*, repeated measures one-way ANOVA; †, data available only for 17 patients; ALT, alanin aminotransferase; AST, aspartate aminotransferase; CK, creatine kinase; CRP, C-reactive protein; Hsp90, Heat shock protein 90; IL, interleukin; LD, lactate dehydrogenase; MCP-1, monocyte chemoattractant protein 1 (CCL2); MIP-1β, macrophage inflammatory protein 1 beta (CCL4); RANTES, regulated on activation/normal T cell expressed and secreted (CCL5); TNF, tumor necrosis factor.

Table S5. Pearson's or Spearman's correlation coefficients of plasma levels of Hsp90 with markers of muscle damage and selected cytokines/chemokines in healthy individuals upon downhill running at selected time intervals

Interval		CK	LD	Myoglobin	AST	ALT	IL-4	IL-7	IL-9	MIP-1β	MCP-1	RANTES	TNF
**baseline**	r	0.459	-0.056	0.039	-0.258	0.554	0.571	-0.124	0.112	0.069	0.424	-0.164	-0.103
	p	**0.048**	0.821	0.875	0.287	**0.014**	**0.033**	0.700	0.658	0.786	0.102	0.515	0.686
	n	18	18	18	18	18	14	12	18	18	16	18	18
**30 min**	r	0.262	0.080	0.489	-0.169	0.309	0.403	-0.292	0.374	0.371	0.598	0.458	0.290
	p	0.278	0.745	**0.034**	0.490	0.198	0.137	0.332	0.127	0.130	**0.011**	0.056	0.243
	n	18	18	18	18	18	15	13	18	18	17	18	18
**1 hour**	r	0.067	-0.148	-0.060	0.210	0.023	-0.101	0.035	0.298	0.284	0.288	0.368	-0.007
	p	0.786	0.544	0.808	0.387	0.925	0.768	0.929	0.229	0.254	0.262	0.132	0.977
	n	18	18	18	18	18	11	9	18	18	17	18	17
**14 days**	r	-0.341	-0.403	-0.328	-0.272	-0.180	-0.125	-0.544	-0.152	-0.082	-0.266	0.055	-0.212
	p	0.153	0.087	0.171	0.261	0.462	0.714	0.084	0.560	0.755	0.302	0.829	0.415
	n	18	18	18	18	18	11	11	17	17	17	18	17
**30 min - baseline**	r	0.058	0.240	0.146	0.316	0.154	0.635	-0.200	-0.016	-0.039	0.385	0.164	0.310
	p	0.814	0.323	0.552	0.188	0.529	**0.020**	0.579	0.950	0.879	0.141	0.515	0.211
	n	18	18	18	18	18	13	9	18	18	16	18	18
**1 hour – baseline**	r	-0.509	-0.261	-0.465	-0.138	-0.475	-0.648	-0.084	-0.214	-0.079	0.362	-0.257	0.051
	p	**0.026**	0.280	**0.045**	0.574	**0.040**	0.269	0.778	0.395	0.754	0.169	0.303	0.844
	n	18	18	18	18	18	11	7	18	18	16	18	17
**30 min – 14 days**	r	-0.050	3.5	0.249	-0.125	0.010	0.685	-0.045	-0.297	-0.317	0.353	-0.162	0.019
	p	0.845	**0.048**	0.320	0.620	0.968	**0.020**	0.916	0.247	0.215	0.165	0.521	0.943
	n	18	18	18	18	18	10	8	18	17	17	18	17
**1 hour – 14 days**	r	0.314	0.153	0.312	0.050	0.093	0.719	0.227	0.040	-0.071	-0.181	-0.082	0.053
	p	0.190	0.533	0.193	0.839	0.704	0.069	0.665	0.880	0.787	0.486	0.748	0.845
	n	18	18	18	18	18	6	6	17	17	17	18	16

Acronyms: Data with normal distribution were assessed using Pearson's correlation coefficient (r). Data with non-normal distribution were assessed using Spearman's correlation coefficient (r). Statistically significant correlations ($p<0.05$) are marked in bold. Hsp90, heat shock protein 90; IIM, idiopathic inflammatory myopathies; IL, interleukin; MCP-1, monocyte chemoattractant protein 1 (CCL2); MIP-1β, macrophage inflammatory protein 1 beta (CCL4); n, number of samples with analyzed parameter; RANTES, regulated on activation/normal T cell expressed and secreted (CCL5); TNF, tumor necrosis factor.
